# Supplementary material for: Tissue and cellular rigidity and mechanosensitive signaling activation in Alexander disease
Source: Nat Commun. 2018 May 15;9:1899. doi: 10.1038/s41467-018-04269-7 (PMC5954157; doi:10.1038/s41467-018-04269-7)
Supplement: Supplementary file 1 — Supplementary Information [file 41467_2018_4269_MOESM1_ESM.pdf]

Supplementary Information

Tissue and cellular rigidity and mechanosensitive signaling activation in Alexander disease

Wang et al.

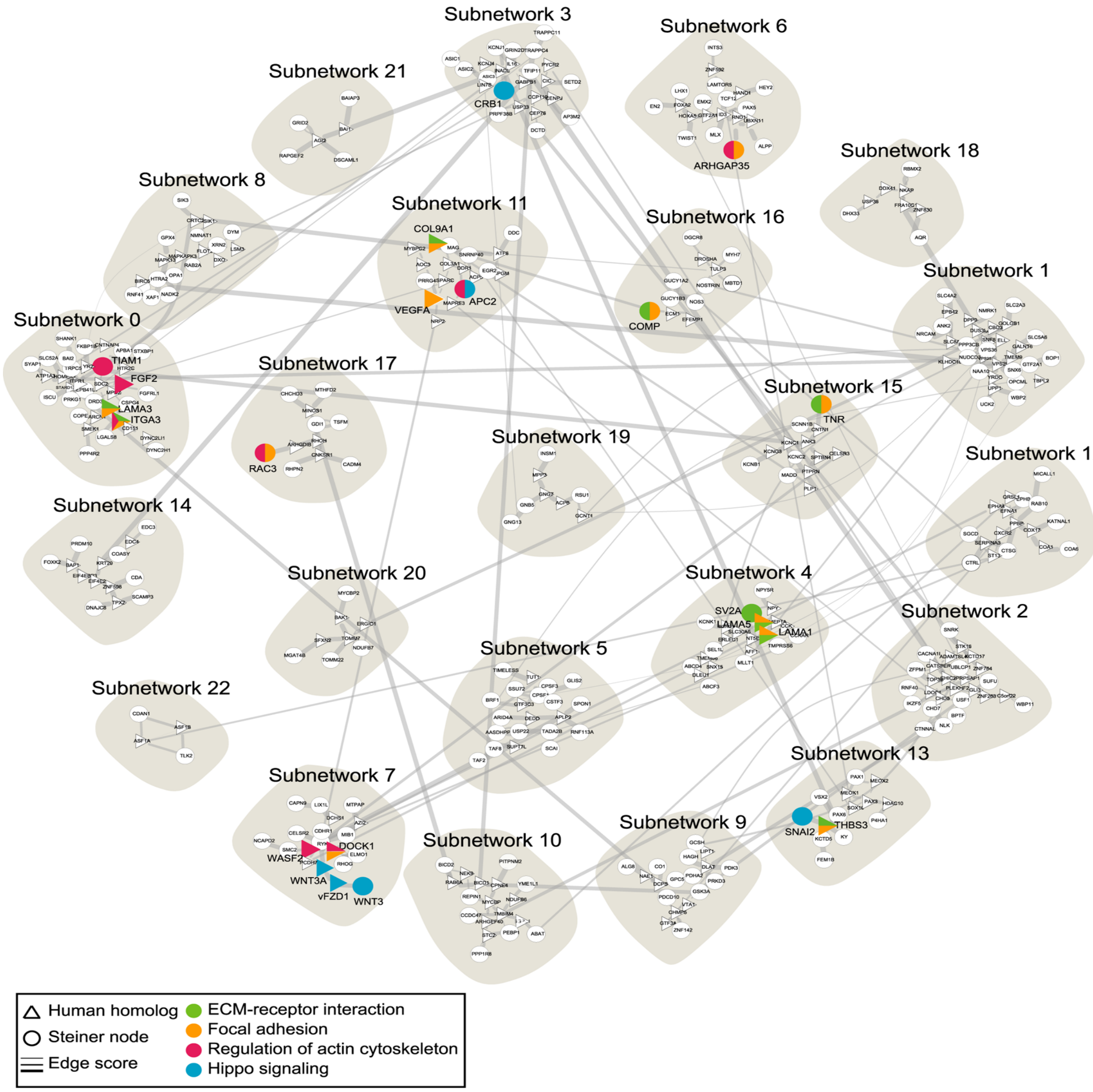

Supplementary Figure 1. Complete overall network solution for the genome-scale genetic screen of Alexander disease. Related to Fig. 1b.

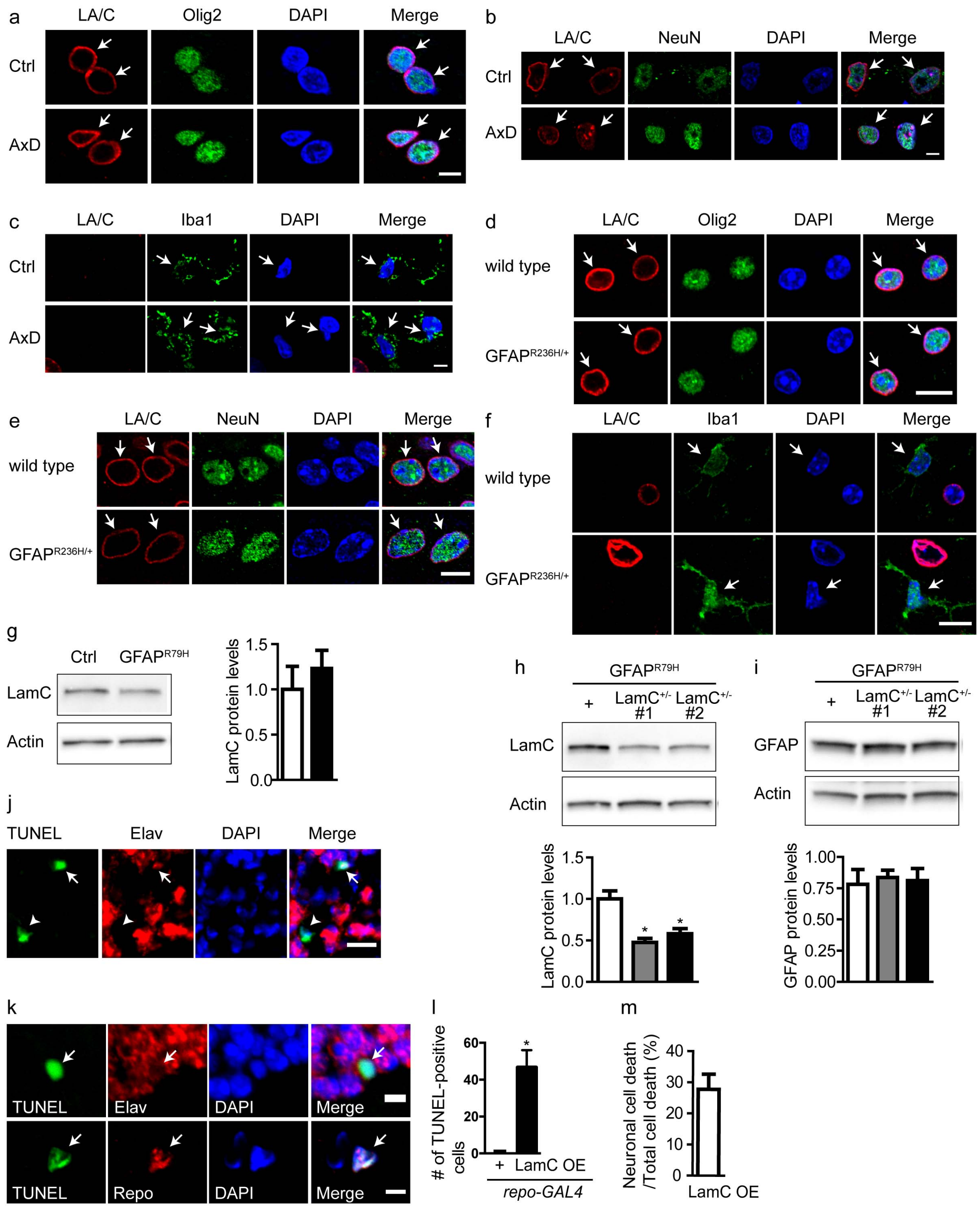

Supplementary Figure 2. a-b Double label immunofluorescence shows similar expression of A-type lamin, lamin A/C (LA/C) in the oligodendrocytes (**a**, arrows) and neurons (**b**, arrows) of a one-year-old Alexander disease patient (AxD) compared to an age-matched control (Ctrl). Olig2 labels oligodendrocytes and NeuN labels neurons. Scale bars are 5 microns. **c** No expression of

lamin A/C was detected in microglial cells of a one-year-old Alexander disease patient (AxD) and an age-matched control (Ctrl). Iba1 labels microglial cells (arrows). Scale bar is 5 microns. **d**-**e** Double label immunofluorescence shows similar expression of A-type lamin, lamin A/C (LA/C) in the oligodendrocytes (**d**, arrows) and neurons (**e**, arrows) of 3-month-old Alexander disease model mice ($GFAP^{R236H/+}$) compared to age-matched wild type littermate mice. Olig2 labels oligodendrocytes and NeuN labels neurons. Scale bars are 10 (**d**) and 5 (**e**) microns. **f** No expression of lamin A/C was detected in microglial cells of 3-month-old Alexander disease model mice ($GFAP^{R236H/+}$) and age-matched wild type littermate mice. Iba1 labels microglial cells (arrows). Scale bar is 10 microns. **g** No significant difference of LamC expression in 1-day-old Alexander disease model flies ($GFAP^{R79H}$) and control flies (Ctrl). N=4. **h** Western blot demonstrates reduced LamC expression in Alexander disease model flies carrying loss-of-function alleles of *LamC*. Flies are 20 days old. * $p<0.05$. Kruskal-Wallis test. **i** Western blot shows equal GFAP levels in Alexander disease model flies carrying loss-of-function alleles of *LamC*. Flies are 20 days old. Blots are reprobed for actin to illustrate equivalent protein loading. **j** Double label immunofluorescence of TUNEL and neuronal cell marker Elav shows an apoptotic neuronal cell (arrow, TUNEL positive and Elav positive) and an apoptotic non-neuronal cell (arrowhead, only TUNEL positive) in 20-day-old Alexander disease model flies ($GFAP^{R79H}$). Scale bar is 5 microns. **k** Double label immunofluorescence of TUNEL and cell type specific markers shows an apoptotic neuronal cell (top row, Elav positive) and an apoptotic glial cell (bottom row, Repo positive) in 10-day-old flies overexpressing LamC in glial cells (*repo-Gal4, UAS-LamC/+*). Scale bars are 2 microns. **l** Quantification of TUNEL-positive cells in 10-day-old flies overexpressing LamC (LamC OE) in glial cells (*repo-Gal4, UAS-LamC/+*). p=0.0022, Mann Whitney test. N=6. **m** Quantification of neuronal cell as a percentage of total cell death in 10-day-old flies overexpressing LamC in glial cells (*repo-Gal4, UAS-LamC/+*). N=6.

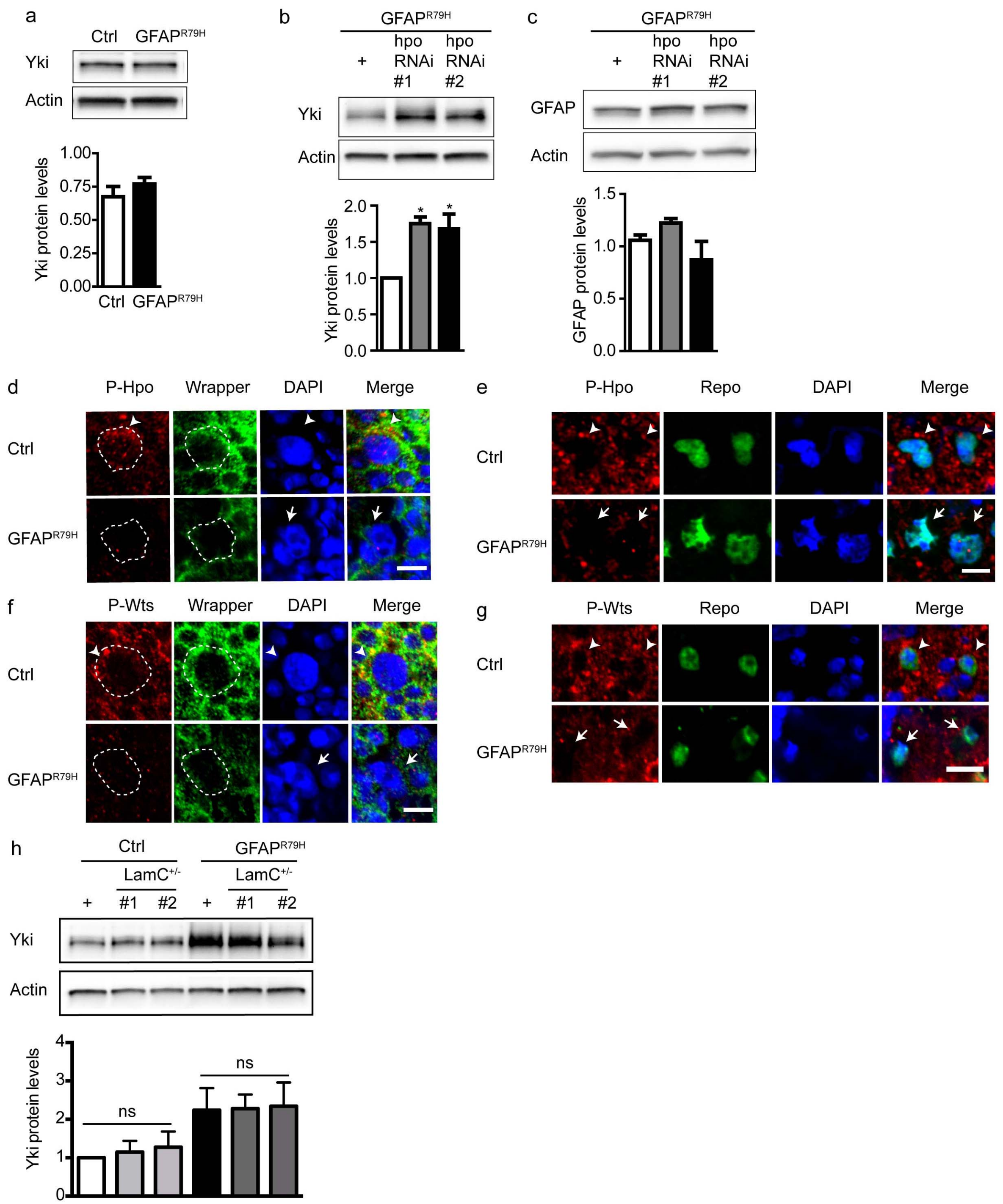

Supplementary Figure 3. a No significant difference of Yki expression in 1-day-old Alexander disease model flies (GFAP^{R79H}) and control flies (Ctrl). N=4. **b** Western blot shows increased Yki expression in Alexander disease model flies carrying transgenic RNAi lines targeting *hpo*. *

$p<0.05$, Friedman test. **c** Western blot demonstrates equal GFAP levels in Alexander disease model flies carrying transgenic RNAi lines targeting *hpo*. **d-e** Double label immunofluoresecence shows reduced phospho-Hpo expression in the glial cells of Alexander disease model flies ($GFAP^{R79H}$, arrows) compared to age-matched controls (top row, arrowheads). Flies are 20 days old. Wrapper labels glial membrane (**d**) which is outlined with white dashed line. Repo marks nuclei of glial cells (**e**). DAPI labels nuclei. Scale bars are 5 microns. **f-g** Double label immunofluorescence shows reduced phospho-Wts expression in the glial cells of Alexander disease model flies ($GFAP^{R79H}$, arrows) compared to age-matched controls (top row, arrowheads). Flies are 20 days old. Wrapper labels glial membrane (**f**) which is outlined with white dashed line. Repo marks nuclei of glial cells (**g**). DAPI labels nuclei. Scale bars are 5 microns. **h** Reducing LamC expression using loss-of-function alleles did not change Yki expression levels in control flies or Alexander disease model flies. Ctrl: *repo-GAL4/+*. $GFAP^{R79H}$: *repo-GAL4, UAS-GFAP*^{R79H}*/+*. Flies are 20 days old. N=4. Blots are reprobed for actin to illustrate equivalent protein loading.

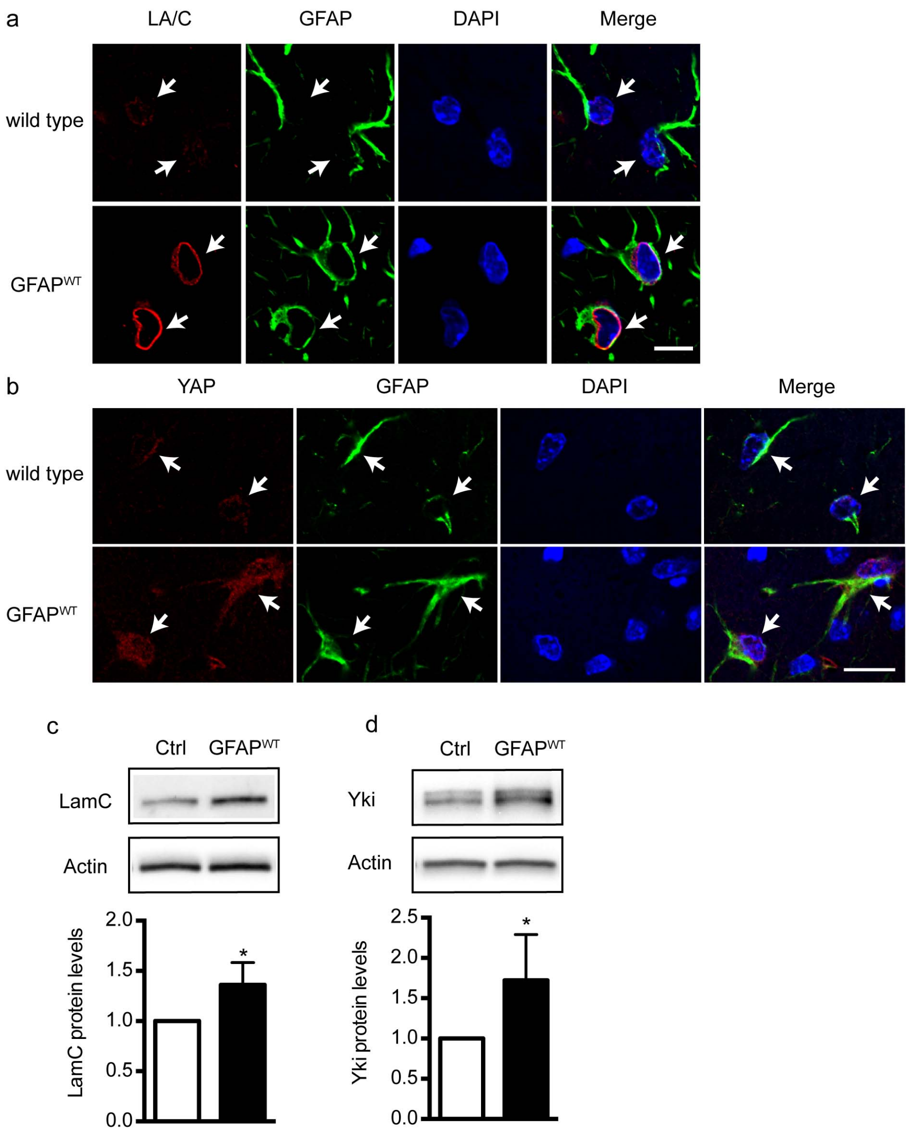

Supplementary Figure 4. a Double label immunofluorescence shows increased lamin A/C (LA/C) expression in the astrocytes of 3-month-old wild type GFAP transgenic mice ($GFAP^{WT}$, arrows) compared to that in age-matched wild type littermate control (wild type, arrows). GFAP labels astrocytes. Scale bar is 10 microns. **b** Double label immunofluorescence shows increased YAP expression in the cytosol and nuclei of astrocytes from 3-month-old wild type GFAP transgenic mice ($GFAP^{WT}$, arrows) compared to that in age-matched wild type littermate control (wild type, arrows). GFAP labels astrocytes. Scale bar is 10 microns. **c** Western blot confirms increased expression of LamC in 20-day-old wild type GFAP transgenic flies ($GFAP^{WT}$)

compared to age-matched control flies. The blot is reprobed for actin to illustrate equivalent protein loading. N=6, p=0.0313, Wilcoxon test. **d** Western blot confirms increased expression of Yki in 20-day-old wild type GFAP transgenic flies ($GFAP^{WT}$) compared to age-matched control flies. The blot is reprobed for actin to illustrate equivalent protein loading. N=6, p=0.0313, Wilcoxon test. Genotypes in **c**,**d**: Ctrl: *repo-GAL4/*+. $GFAP^{WT}$: *repo-GAL4, UAS-*$GFAP^{WT}$/+.

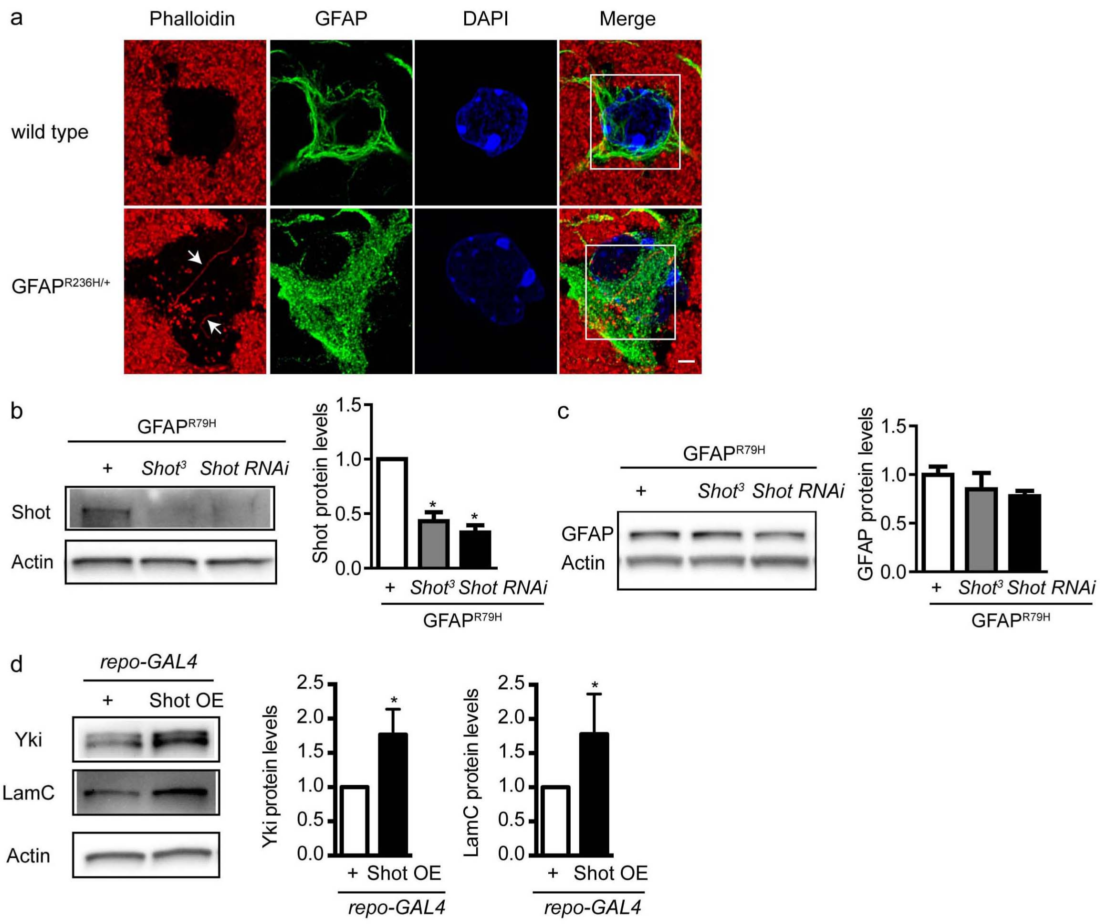

Supplementary Figure 5. a Whole cell view shows F-actin bundles (arrows, bottom panel) in the astrocytes of 3-month-old Alexander disease model mice, but not in age-matched control mice. Phalloidin labels F-actin. GFAP marks astrocytes. DAPI labels nuclei. Scale bar is 2 microns. White box marks region of interest shown in Fig. 4a. **b** Western blot demonstrates reduced Shot expression in Alexander disease model flies carrying loss-of-function alleles of *shot*. Flies are 20 days old. N=5. $p<0.05$, Friedman test. **c** Western blot shows similar GFAP levels in Alexander disease model flies carrying loss-of-function alleles of *shot*. Flies are 1 day old. N=3. **d** Western blots confirm increased Yki and LamC expression in 20-day-old flies expressing Shot (*repo-GAL4, UAS-shot*; Shot OE for simplicity). Blots are reprobed for actin to illustrate equivalent protein loading. N=6, p=0313 (Yki) and N=8, p=0.0078 (LamC), Wilcoxon test.

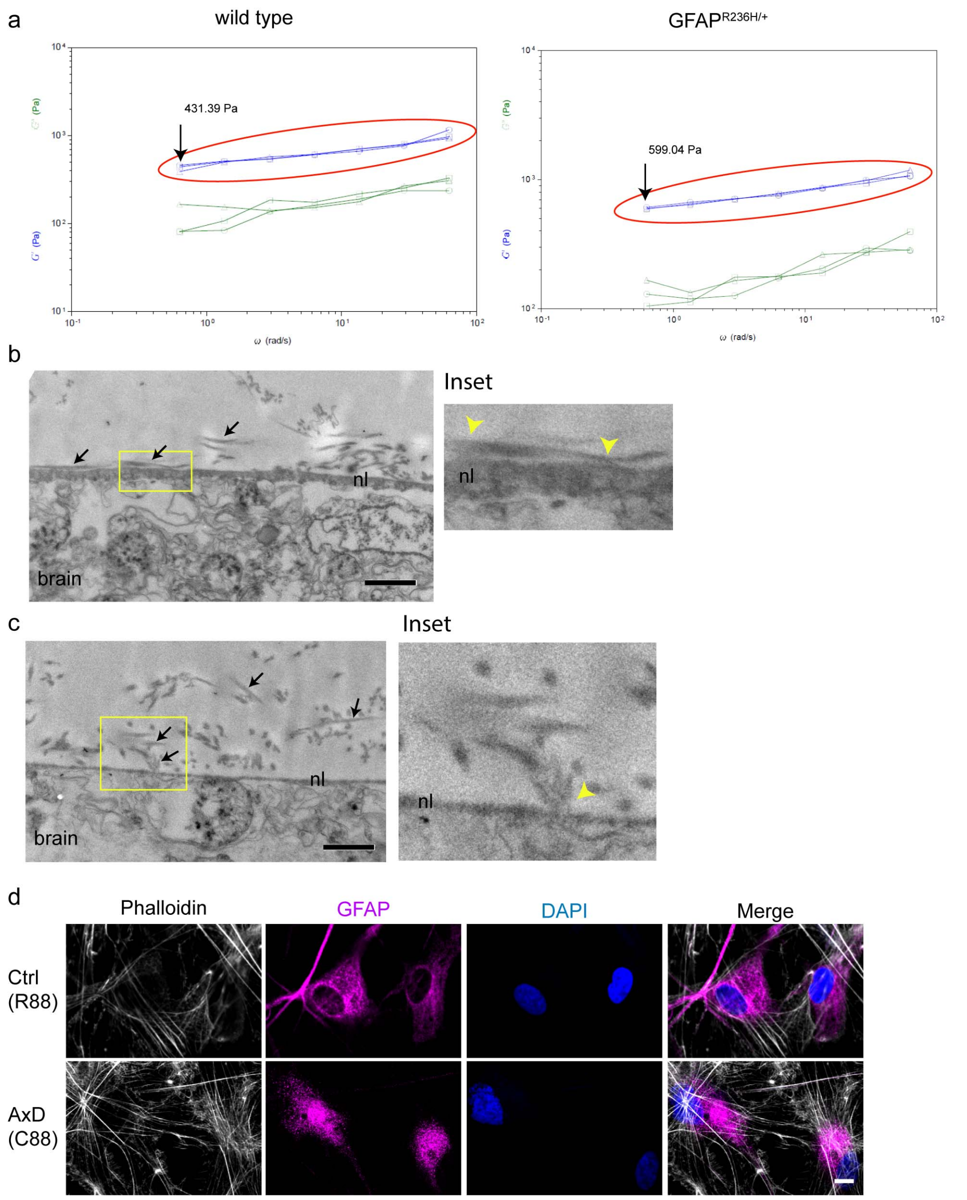

Supplementary Figure 6. a Examples of rheometer measurement graph in Alexander disease model mice (*GFAP*$^{R236H/+}$) and age-matched wild type littermate control (wild type). Storage modulus (G') was swept through 7 different frequencies (0.1 Hz to 10 Hz) and the value at the lowest frequency (0.1 Hz, arrows) was used for quantification. **b-c** Two representative electron microscopic images show collagen fibrils (arrows and arrowheads in insets) contacting the neural lamella (nl), the collagen-rich outer extracellular matrix layer of the *Drosophila* brain.

Genotype: *repo-GAL4/+*. Scale bars are 1 micron. **d** Stress fibers in astrocytes differentiated from iPS cells of Alexander disease patient and control. Individual, unmerged panels from the composite presented in Fig. 6c. Phalloidin labels F-actin. GFAP marks astrocytes. DAPI labels nuclei. Scale bar is 10 microns. Expanded view for Fig. 6c.

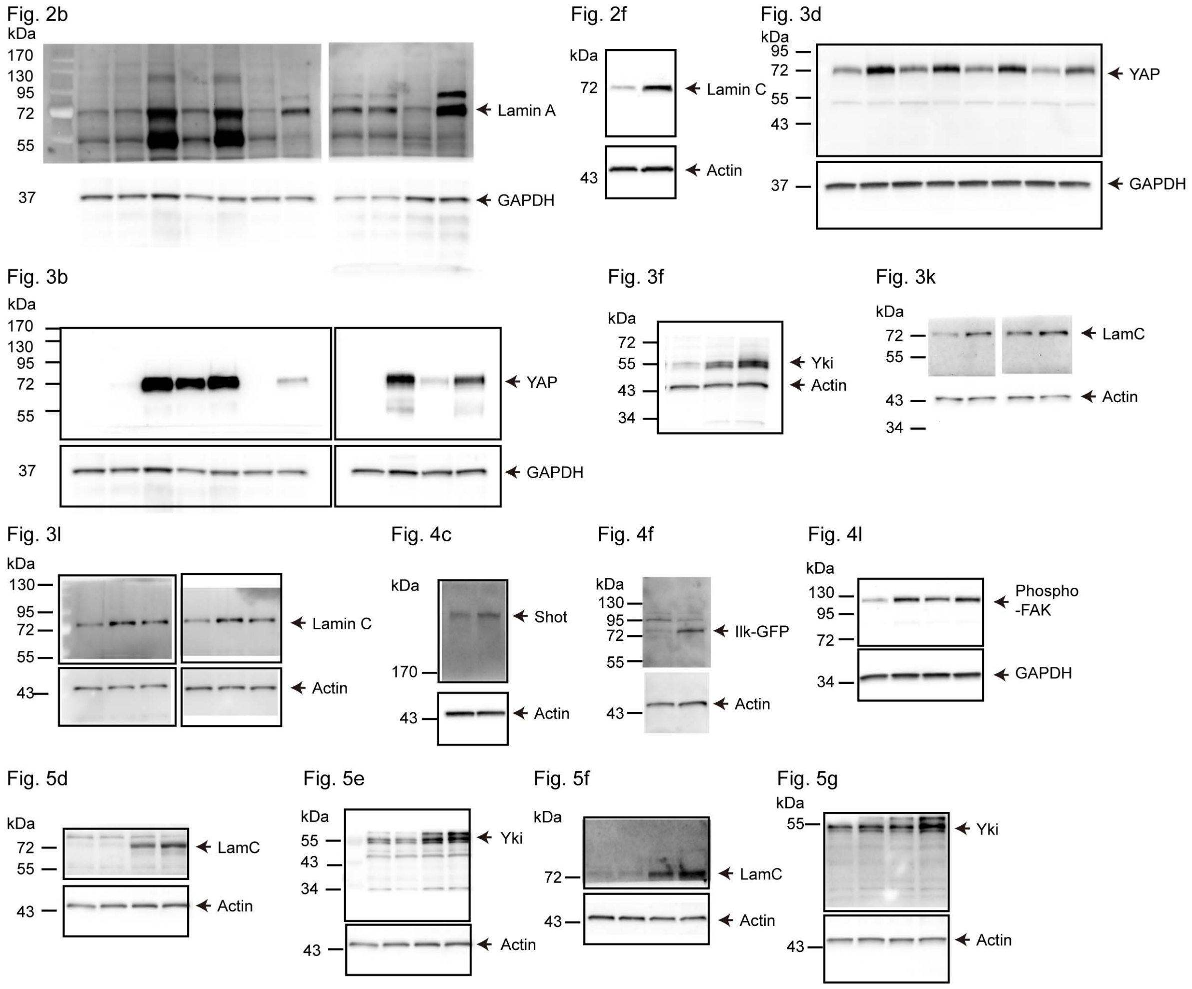

Supplementary Figure 7. Full length blots showing molecular weights for main figures.

Supplementary Table 1. KEGG pathway analysis implicates mechanotransduction pathways.

**Term**	**Adjusted P-value**	**Genes**
ECM-receptor interaction_Homo sapiens_hsa04512	3.4474E-06	COMP;LAMA5;ITGA3;SV2A;LAMA1; LAMA3;COL9A1;TNR;THBS3
Focal adhesion_Homo sapiens_hsa04510	1.89289E-05	COMP;LAMA5;ITGA3;LAMA1;LAMA3;COL9A1; RAC3;TNR;DOCK1;ARHGAP35;VEGFA;THBS3
Regulation of actin cytoskeleton_Homo sapiens_hsa04810	0.006785522	APC2;TIAM1;ITGA3;RAC3;FGF2;DOCK1; WASF2;ARHGAP35
Hippo signaling pathway_Homo sapiens_hsa04390	0.014205577	FZD1;APC2;CRB1;WNT3A;SNAI2;WNT3
