# Supplementary material for: Tissue and cellular rigidity and mechanosensitive signaling activation in Alexander disease
Source: Nat Commun. 2018 May 15;9:1899. doi: 10.1038/s41467-018-04269-7 (PMC5954157; doi:10.1038/s41467-018-04269-7)
Supplement: Supplementary file 3 — Description of Additional Supplementary Files [file 41467_2018_4269_MOESM3_ESM.pdf]

Description of Additional Supplementary Files:

Supplementary Data 1: This data set is the summary of the genome-wide genetic screen in the *Drosophila* model of Alexander disease. Sheet 1 lists all the transgenic RNAi lines tested. Sheet 2 and Sheet 3 list suppressors and enhancers identified in the screen. Related to **Fig. 1** and **Supplementary Fig. 1.**

Supplementary Movie 1: This movie is a three-dimensional (3D) reconstruction of F-actin bundles (Red) and GFAP (Green) in the astrocyte of a 3-month-old Alexander disease model mouse ($GFAP^{R236H/+}$). Related to **Fig. 4a** and **Supplementary Fig. 5a.**
